# Autophagy–ferroptosis crosstalk in sepsis: metabolic pathways, redox injury, and host-directed antioxidant nanomedicine

**DOI:** 10.3389/fimmu.2026.1855630

**Published:** 2026-05-28

**Authors:** Yang Huang, Shi Feng, Jiaqi Wang, Fan Yi, Yuhong Gao

**Affiliations:** Central Hospital Affiliated to Shenyang Medical College, Shenyang, Liaoning, China

**Keywords:** antioxidant nanomedicine, autophagy, ferroptosis, host-directed therapy, immunometabolism, lipid peroxidation, oxidative stress, sepsis

## Abstract

Sepsis is a dynamic syndrome of infection-driven metabolic and immune dysregulation in which oxidative stress can escalate into an “oxidative storm,” promoting organ dysfunction and maladaptive host responses. Within this context, ferroptosis represents a metabolically constrained form of regulated necrotic cell death driven by iron-dependent lipid peroxidation, linking redox collapse to tissue injury in sepsis. Emerging evidence suggests that autophagy critically shapes ferroptosis susceptibility by regulating intracellular iron mobilization, membrane lipid substrate availability, mitochondrial quality control, and energy-stress signaling. This review therefore frames autophagy–ferroptosis crosstalk in sepsis as a host metabolic vulnerability and discusses how mechanism-guided, host-directed antioxidant nanomedicine may help preserve tissue integrity while limiting interference with antimicrobial defense. We explored how autophagy modulates ferroptosis susceptibility by regulating iron metabolism, lipid substrate availability, and mitochondrial quality control. Building on this framework, we evaluated emerging antioxidant nanomedicines targeting key intervention points, including iron chelation, catalytic ROS/RNS scavenging, membrane-localised radical trapping, mitochondria-targeted source control, and enhancement of endogenous defences. Organ- and immune-specific effects are highlighted, emphasizing the need for aligned biochemical readouts, flux-aware autophagy evaluation, and stage-specific therapeutic targeting. Finally, we outline translational priorities for precision redox modulation in sepsis, focusing on biomarker-guided patient stratification, compartment-specific delivery, and biosafety considerations.

## 1 Introduction

Sepsis remains a major global health burden and is the leading cause of preventable death (1). It is defined as life-threatening organ dysfunction caused by a dysregulated host response to infection (2). Although current standard-of-care management has improved early recognition and supportive care, mortality rates remain unacceptably high (1). Poor outcomes in sepsis are driven not only by failure to control infection, but also by profound and progressive immune dysregulation (2). During sepsis, the host immune response frequently transitions from early hyperinflammation to profound immunosuppression or immunoparalysis (2). This transition increases the risk of secondary nosocomial infections and drives persistent multi-organ failure (3). Effective therapeutic interventions should target the core mechanisms of parenchymal tissue injury while rigorously preserving essential antimicrobial defenses, rather than relying solely on broad immunosuppression or pathogen eradication (1).

Redox dysregulation is a fundamental hallmark of sepsis (1, 4). During infection, oxidative stress frequently escalates into an “oxidative storm”—a sustained, self-reinforcing overload of reactive oxygen species (ROS) and reactive nitrogen species (RNS), coupled with severely impaired redox buffering and the pathological expansion of redox-active labile iron pool (LIP) (1, 3, 5). This toxic biochemical environment actively promotes lipid peroxidation and engages regulated cell death programs, causing organ failure (3). Among these pathways, ferroptosis, an iron-dependent form of lipid peroxidation-driven cell death, is highly relevant to sepsis pathology (3, 6). Under severe septic stress, intense oxidant pressure rapidly depletes endogenous reductive capacity and severely compromises primary ferroptosis defense mechanisms, most notably the glutathione peroxidase 4 (GPX4)–glutathione (GSH) axis (2, 3). Once these defenses fail, lipid hydroperoxides accumulate and disrupt membrane integrity (3). Highly metabolic organs with immense ATP demands and extensive lipid bilayer networks, such as the heart and kidneys, are particularly vulnerable to redox-driven injury (6).

Ferroptosis is not a simple consequence of generalized ROS elevation (3). It is tightly regulated by the convergence of iron metabolism, membrane lipid remodeling, generation of mitochondrial reactive oxygen species (mtROS), and endogenous antioxidant defenses (3, 7). The GPX4–GSH axis is the primary enzymatic defense against lipid peroxidation (3, 7). Additional systems can also modulate ferroptosis susceptibility, including ferroptosis suppressor protein 1-mediated coenzyme Q (CoQ) reduction at the plasma membrane, the dihydroorotate dehydrogenase–CoQ axis in mitochondria, and GTP cyclohydrolase 1 (GCH1)–tetrahydrobiopterin (BH_4_) signaling (3, 7, 8). In sepsis, these pathways should be interpreted in a context-specific manner, as ferroptosis susceptibility is tightly coupled to autophagy-dependent metabolic reprogramming (2, 3, 6). Autophagic flux exerts rigorous control over intracellular iron mobilization, lipid substrate availability, and targeted clearance of damaged mitochondria (6). This crosstalk between autophagy and ferroptosis underscores a key therapeutic challenge in sepsis management, the “antioxidant paradox” (1). Late-stage oxidative stress unequivocally drives multi-organ failure, and early bursts of ROS and RNS are vital for innate immune pathogen clearance (1). To successfully protect the organ tissue without compromising host immunity, redox interventions should be strictly confined to the subcellular sites of injury at the appropriate disease stage (4).

Translating this demand for spatiotemporal precision into viable clinical therapies remains challenging (4). Although traditional small-molecule inhibitors, such as ferrostatin-1, have shown strong proof-of-concept in preclinical studies, their clinical utility remains limited by unfavorable pharmacokinetics and insufficient subcellular targeting (4, 9, 10). Poor aqueous solubility, rapid metabolic clearance, and non-specific biodistribution restrict their accumulation within the lipid bilayers and mitochondrial compartments, where ferroptotic chemistry is most likely to propagate (4, 9, 10).

Engineered nanomedicine solutions may help overcome pharmacokinetic and targeting barriers (4, 9). In addition to enhancing the blood circulation time of lipophilic antioxidants, advanced nanotherapeutics can function as engineered exogenous redox-control systems that integrate targeted delivery with localized catalytic or iron-buffering activity (4, 9, 10). Rather than merely encapsulating traditional drugs, these nanoscale platforms integrate precise therapeutic delivery with intrinsic physicochemical properties, such as catalytic ROS neutralization or localized iron chelation (9, 10). By actively targeting specific subcellular sites of injury, these engineered nanomedicines can block the iron ignition, lipid propagation, and mitochondrial amplification axes of ferroptosis while preserving systemic immune boundaries (4).

This review presents a mechanism-driven framework for deploying precision antioxidant nanotherapeutics in sepsis, with emphasis on compartment-restricted delivery, aligned biochemical validation, and stage-aware redox control (9, 10). We first outline the biochemistry of the oxidative storm, tracing the precise sequence from LIP to terminal lipid peroxidation and ferroptotic cell death (3). By mapping the crosstalk between autophagy and ferroptosis, we identified therapeutic targets and corresponding biochemical markers required to confirm these mechanisms (6). We categorized emerging nanomedicines based on their subcellular sites of action: membrane-targeted radical-trapping antioxidants (RTAs), catalytic nanozymes, metal–phenolic networks for targeted iron chelation, and mitochondria-directed source control (9, 10). Finally, we evaluate the translational roadmap, emphasizing the necessity of biomarker-guided patient stratification, subcellular targeting, and strict adherence to immune biosafety constraints to resolve the antioxidant paradox and achieve true organ protection (1, 4).

## 2 Oxidative storm and ferroptosis in sepsis

**Figure 1:**
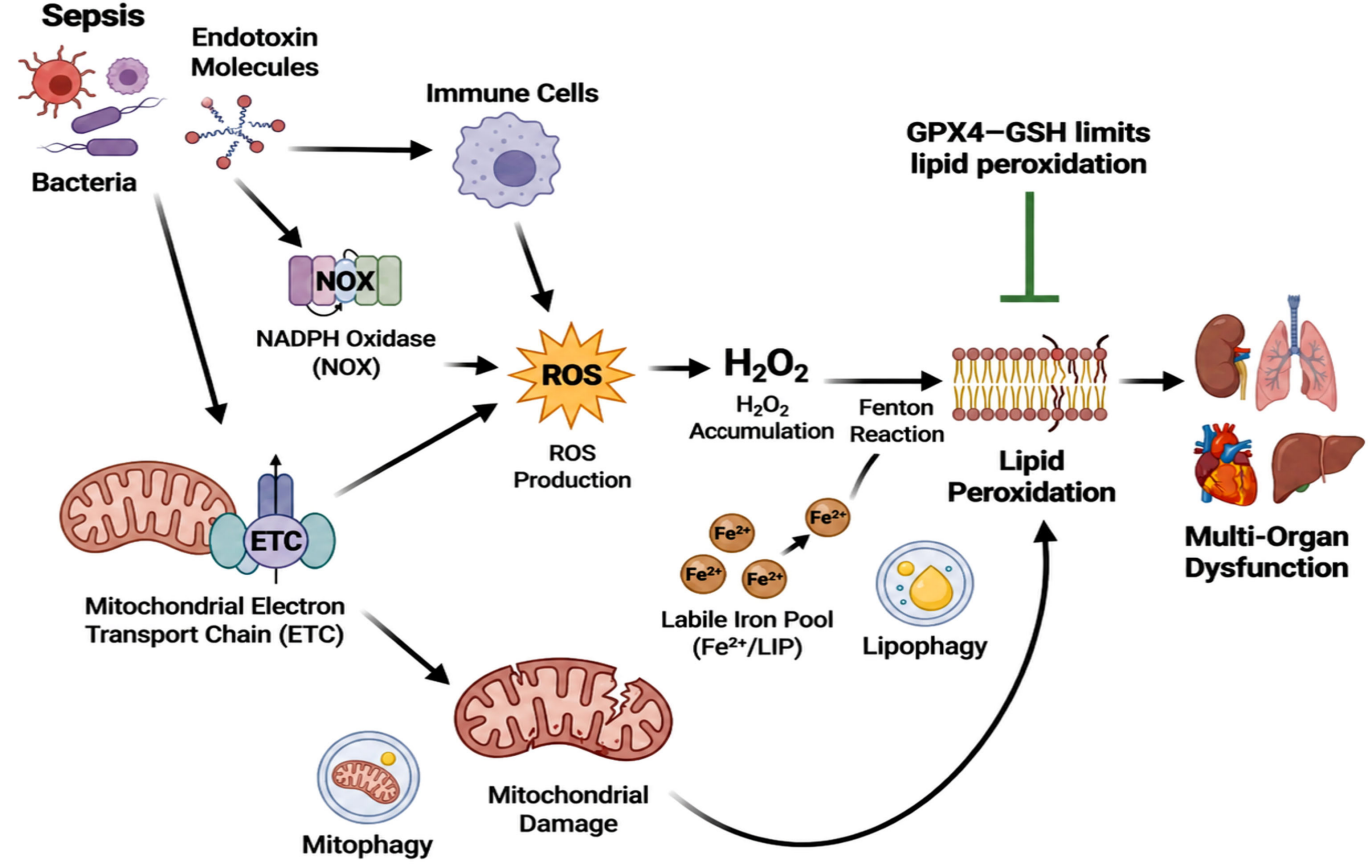
The mechanistic framework of the oxidative storm and ferroptosis in sepsis. Sepsis-induced immune activation and mitochondrial damage lead to the excessive production of reactive oxygen species (ROS) via NADPH oxidase (NOX) and the mitochondrial electron transport chain (ETC). Superoxide is rapidly converted to hydrogen peroxide (H_2_O_2_), which subsequently encounters an expanded labile iron pool (LIP) to trigger the Fenton reaction. This iron-dependent ignition drives the accumulation of lipid peroxides, ultimately leading to multi-organ dysfunction (e.g., kidneys, lungs, heart, liver). Dysregulated autophagic pathways (mitophagy, lipophagy, and ferritinophagy-mediated iron release) further modulate ferroptosis susceptibility. The glutathione peroxidase 4–glutathione (GPX4–GSH) axis serves as the primary endogenous defense gate to limit lipid peroxidation propagation. Created in BioRender. Huang, Y. (2026) https://BioRender.com/4nq55fc.

In sepsis, strained redox control can escalate into an “oxidative storm” that biases cells toward ferroptosis by promoting iron-dependent ignition, lipid-peroxidation propagation, and failure of endogenous defense gates (3, 5, 11). When an expanded LIP is combined with elevated hydrogen peroxide (H_2_O_2_), Fenton chemistry initiates lipid peroxidation reactions that can propagate through polyunsaturated fatty acid (PUFA)-containing membranes (3, 12, 13). The ferroptosis defense gates may then be overwhelmed, allowing lipid hydroperoxides to accumulate and drive ferroptotic injury (3, 5). The major redox and iron-dependent events linking sepsis-associated oxidative stress to lipid peroxidation, ferroptosis susceptibility, and multi-organ dysfunction are summarized in **Figure 1**.

### 2.1 Mitochondrial and NADPH oxidase-derived H_2_O_2_ in sepsis

Sepsis triggers rapid overproduction of ROS and RNS that can quickly overwhelm intrinsic antioxidant capacity (1, 14). The two major sources of this oxidative stress are electron leakage from the mitochondrial electron transport chain (ETC) and activation of NADPH oxidase (NOX) complexes (5, 11, 15). Additional contributions arise from inducible nitric oxide synthase (iNOS), myeloperoxidase-dependent reactions in neutrophils, and dysregulated endothelial redox enzymes (14, 16). During sepsis, inflammatory signaling, microcirculatory hypoxia, and shifts in substrate utilization impair mitochondrial function and increase superoxide generation (11, 15). NOX complexes are activated in immune cells and inflamed parenchymal tissues (16, 17). Although NOX-derived superoxide is essential for early antimicrobial defense, persistent activation increases systemic oxidative stress and causes tissue injury (16, 17).

Superoxide from both mitochondrial and NOX-derived sources is rapidly converted to H_2_O_2_ by superoxide dismutase (SOD) (14, 15). Unlike highly reactive but short-lived free radicals, H_2_O_2_ is relatively stable and membrane-permeable (2, 14). Under septic stress, it can accumulate and promote Fenton chemistry when redox-active iron is available (2, 14). In this setting, peroxide accumulation drives lipid peroxidation and increases susceptibility to ferroptotic injury (2, 11).

RNS biology also defines an important boundary for antioxidant strategies in sepsis (1, 14). Nitric oxide is required for vascular regulation and pathogen clearance (16, 17). However, excessive nitrative stress impairs mitochondrial function, disturbs thiol redox control, and modifies iron-sulfur clusters, thereby reshaping intracellular iron availability and the surrounding redox environment (14, 15, 17). Together, these processes show that oxidative injury in sepsis is not simply a matter of generalized ROS excess, but reflects the convergence of mitochondrial dysfunction, NOX activation, peroxide accumulation, iron dysregulation, and RNS-mediated redox remodeling (1, 2, 11, 14–19).

### 2.2 Iron dysregulation and fenton ignition

During sepsis, disrupted iron handling allows elevated H_2_O_2_ to coincide with expanded intracellular pools of redox-active iron. These conditions favor Fenton ignition, lipid peroxidation, and ferroptotic injury (3, 11, 12).

LIP expansion is a central upstream change (2, 3). Under physiological conditions, iron is tightly controlled and largely stored in ferritin (14). Sepsis disrupts iron trafficking and storage (12). Under hemolytic and heme-stress conditions, macrophages phagocytose damaged erythrocytes, thereby increasing intracellular ferrous iron (Fe²^+^) (11). Lysosome-dependent processing of iron-containing cargo, such as ferritinophagy, further expands the LIP (6).

When H_2_O_2_ is available, Fe²^+^ catalyzes the decomposition of H_2_O_2_ to produce highly reactive hydroxyl radicals (•OH) (2, 3, 5). These oxidants abstract hydrogen atoms from PUFA-containing membrane phospholipids, thereby initiating lipid peroxidation (2, 3). Once initiated, this process continues to drive membrane damage, independent of the initial ROS burst (3). This helps explain why ferroptotic injury can rapidly accelerate when abundant H_2_O_2_ coincides with an expanded LIP (3, 11, 12).

### 2.3 Lipid peroxidation initiation and propagation

Lipid peroxidation is the core process of ferroptosis and a key feature that distinguishes it from other regulated cell death programs (2, 3). It starts from localized oxidation but spreads within membranes through chain propagation, converting local damage into broader membrane dysfunction (13, 20). Non-enzymatic autoxidation drives this lipid-peroxidation chain chemistry (21), while enzyme-linked processes modulate substrate supply and peroxide formation (22, 23).

Once Fenton ignition begins, highly reactive oxidants abstract bis-allylic hydrogen atoms from polyunsaturated fatty acid-containing phospholipids (PUFA-PLs) (21, 24). This generates phospholipid intermediates, which rapidly react with molecular oxygen to form lipid peroxyl radicals (2, 5, 20). Under septic oxidant and metabolic conditions, PUFA-PL oxidation can overwhelm detoxification capacity, thereby accelerating ferroptotic tissue damage (3, 22).

Lipid peroxyl radicals then remove hydrogen atoms from adjacent PUFA-PLs, thereby generating new oxidizable sites and perpetuating lipid peroxidation (20, 21). Repeated oxidative cycles drive lipid hydroperoxide accumulation in plasma and organelle membranes, disrupting bilayer structure and lipid packing (2). As this burden rises, membrane integrity and fluidity decline, permeability increases, and organelle function deteriorates; in extreme cases, membranes rupture and cells undergo necrotic-like death (3, 22). This propagation-dominant behavior explains why RTAs can be effective: they intercept reactive intermediates within the lipid bilayer and halt the propagation cycle, rather than relying only on lowering upstream oxidant pressure (13, 20).

Enzymatic networks may further shape lipid peroxidation through two broad routes (3, 23). First, lipid remodeling enzymes alter the membrane’s lipid composition to actively enrich peroxidation-prone PUFA-PL substrates (22). Second, specialized oxidant enzymes actively catalyze lipid peroxide generation under oxidative stress (25). Under these conditions, cell survival depends on the integrity of the endogenous antioxidant defense system.

### 2.4 Endogenous defense gates and their failure

Ferroptotic injury in sepsis reflects not only oxidant stress but also the capacity of endogenous “gates” that normally detoxify lipid peroxides (3, 5). The GPX4–GSH axis serves as the primary enzymatic defense against lipid peroxidation (3, 7). GPX4 limits ferroptosis by reducing lipid hydroperoxides to stable lipid alcohols, thereby curbing chain propagation (7, 13).

In sepsis, multiple intersecting stressors can simultaneously weaken this axis (5, 26, 27). Persistent ROS/RNS burden consumes GSH (14), while metabolic stress and disrupted substrate handling can limit its intracellular regeneration (15). Under these conditions, lipid hydroperoxides accumulate faster than can be removed by GPX4-dependent detoxification (5).

Beyond the GPX4–GSH axis, additional ferroptosis-resistant pathways have been described as GPX4-independent layers that can buffer lipid-radical stress in specific compartments (28, 29). The ferroptosis suppressor protein 1 (FSP1)–CoQ system is commonly framed as a membrane-associated radical-trapping pathway that continuously regenerates ubiquinol (CoQH_2_) to intercept propagating lipid intermediates (7, 29). DHODH is linked to CoQ reduction within mitochondria and is often recognized as a mitochondrial safeguard (3, 7, 30). In sepsis, these pathways can act as auxiliary safeguards that may modulate susceptibility when the primary GPX4 gate is under strain (3). The protective reliance is highly context-dependent across different tissues and disease stages, changes in their protein expression alone are insufficient to confirm functional ferroptotic mechanisms (3).

### 2.5 Timing, immune boundary, and the antioxidant paradox

Sepsis is an inherently dynamic syndrome, with profound transitions in cellular redox as the disease progresses (1, 31). Early phases often rely on reactive species biology for pathogen control, including oxidative bursts within antimicrobial compartments (14, 32). Later phases are characterized by persistent oxidative injury, metabolic failure, and immunoparalysis (33). This time dependence explains why the same redox-directed intervention can be protective in one context but detrimental in another (1).

In sepsis, the “antioxidant paradox” reflects this stage-dependent contrast (5, 15): reactive species support host defense, but sustained redox stress also drives tissue injury (31). The therapeutic objective is not to avoid antioxidants but to avoid indiscriminate redox suppression. Targeted redox interventions are clinically viable only when deployed in a stage-specific and spatially restricted manner, protecting vulnerable tissues and organelles while limiting interference with sepsis-relevant antimicrobial effector functions (1, 5).

## 3 Autophagy–ferroptosis crosstalk in sepsis

In sepsis, ferroptosis cannot be explained solely by generalized oxidative stress (3). Instead, it arises from the convergence of LIP expansion (34, 35), enrichment of peroxidation-prone membrane lipids (36), and mitochondrial injury. Autophagy reduces cellular injury by removing damaged organelles and oxidized materials. However, when this clearance process becomes dysregulated, it can increase ferroptotic vulnerability by expanding redox-active iron availability or shifting lipid pools toward more oxidizable substrates (35, 36).

**Figure 2:**
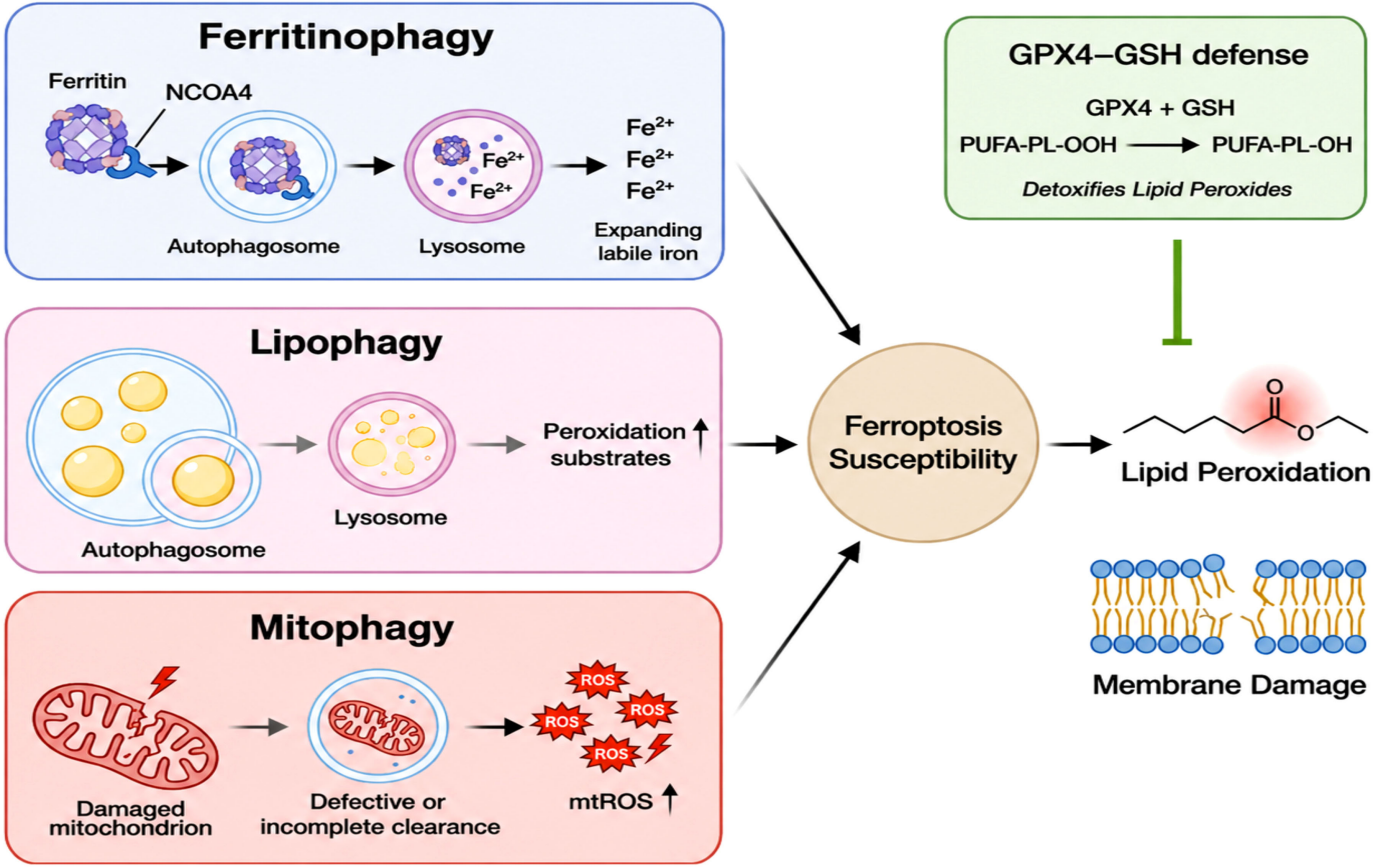
Autophagy-dependent modulation of ferroptosis susceptibility in sepsis. Dysregulated autophagic pathways exacerbate ferroptotic injury through three intersecting axes. Ferritinophagy, mediated by the nuclear receptor coactivator 4 (NCOA4) receptor, routes ferritin to lysosomes, thereby expanding the labile iron pool (LIP). Lipophagy mobilizes lipid droplets, increasing the availability of peroxidation-prone substrates. Defective mitophagy fails to clear damaged mitochondria, resulting in the accumulation of mitochondrial reactive oxygen species (mtROS). Together, these pathways increase cellular susceptibility to ferroptosis, ultimately driving lipid peroxidation and membrane damage when the endogenous glutathione peroxidase 4–glutathione (GPX4–GSH) defense capacity is overwhelmed. Created in BioRender. Huang, Y. (2026) https://BioRender.com/4nq55fc.

Autophagy is often judged from single isolated static protein markers, but sepsis frequently disrupts lysosomal acidification, vesicle trafficking, and cellular energetics; therefore, autophagosomes often accumulate even as lysosomal degradation becomes inefficient (37–39). Autophagosomes may accumulate despite inefficient lysosomal degradation, so an apparent increase in autophagic structures may reflect impaired clearance rather than enhanced cellular protection (40). Whether lysosomes function as productive clearance compartments or retain iron- and lipid-rich cargo depends on autophagic flux (3). **Figure 2** summarizes the major autophagy-dependent mechanisms that shape ferroptosis susceptibility in sepsis, including ferritinophagy, lipophagy, mitophagy impairment, and GPX4–GSH-mediated restraint of lipid peroxidation.

### 3.1 Ferritinophagy and lysosomal iron mobilization

As described in Section 2, the septic oxidative storm expands the intracellular LIP (5, 14). Autophagy contributes to this remodeling because ferritin breakdown is lysosome-dependent (34). Under physiological conditions, ferritin can safely sequester iron in a biochemically stable state. However, the specialized autophagic process of ferritinophagy, directed by the precise cargo receptor NCOA4, routes ferritin to lysosomes for degradation, thereby releasing free iron back into the intracellular LIP (34, 35, 41). In the highly oxidative microenvironment of sepsis, even modest increases in chelatable Fe²^+^ can increase hydroxyl-radical formation via Fenton chemistry and favor the initiation of lipid peroxidation (5, 6, 42).

The clinical objective is not simply to “block ferritinophagy,” but rather to precisely manage iron mobilization amid the oxidative storm (41, 42). Lysosomal competence is a key constraint (14). When septic stress disrupts or functionally impairs lysosomes, autophagic iron release becomes chaotic and poorly controlled, directly fueling Fenton ignition (6, 34). If lysosomes remain functional, iron handling is more likely to be coupled to re-sequestration and redox buffering rather than uncontrolled release (35).

Interventions are more likely to succeed when redox control is subcellularly targeted. Lysosome-local strategies, such as iron chelation and microenvironment-restricted peroxide buffering, may help decouple lysosomal iron mobilization from Fenton ignition (8, 34). By lowering local H_2_O_2_ burden in the same compartment as redox-active iron, these approaches can limit downstream lipid peroxidation and thereby reduce ferroptotic injury (42). Mechanistic support should rely on iron-facing measures together with lipid peroxidation readouts rather than autophagic protein markers alone (6).

### 3.2 Lipophagy and membrane lipid remodeling

Ferroptosis is mediated by lipid peroxidation (2, 3). This depends on the availability and distribution of peroxidation-prone lipid substrates in membranes (36). Autophagy contributes to this substrate through lipophagy and lipid mobilization, shaping membrane vulnerability in ways that are easily overlooked in sepsis-focused literature (3, 36, 43).

PUFA-PLs supply the substrate pool that sustains lipid peroxidation chain reactions (2, 3, 5). Lipophagy-linked processes actively shape membrane PUFAs by mobilizing lipid stores and directing fatty acids toward phospholipid synthesis (3, 36). In ferroptosis, PUFA-PL enrichment is commonly linked to remodeling enzymes, such as ACSL4 and LPCAT3 (22, 44–46), whereas alternative lipid compositions are generally associated with relative resistance (45). In sepsis, these remodeling pressures are amplified by oxidative stress and metabolic instability. This metabolic failure actively enriches cellular membranes with oxidizable lipids when endogenous antioxidant defense gates are compromised (3, 22).

This membrane lipid composition shapes propagation once lipid radical initiation occurs. Propagation efficiency depends in part on how closely PUFA-PLs are packed because this spatial density affects the chain-transfer probability (36). Lipid remodeling also influences membrane organization and repair capacity, which in turn affects whether oxidized lipids are removed or persist for continued propagation (36, 47). RTAs are designed to intercept lipid peroxidation chains within the hydrophobic core, where chain reactions amplify membrane damage (5). This keeps the intervention focused on the membrane step itself rather than relying on broad upstream ROS suppression. It also fits the sepsis setting, where autophagy-linked lipid handling can reshape the pool and local distribution of oxidizable lipids, creating conditions in which lipid peroxidation is more likely to become self-sustaining (3, 22).

### 3.3 Mitophagy and mtROS amplification

Mitochondria serve as both targets and amplifiers in sepsis (11, 48). As they sustain structural injury, mitochondria increase electron leakage and elevate mtROS output (5, 14, 39). The resulting mtROS further damages mitochondrial structure and function, creating a reinforcing loop that sustains oxidant pressure (11, 49).

mtROS does not merely contribute to general oxidative burden (8). It amplifies ferroptosis through three coupled effects: increasing lipid peroxidation pressure, accelerating intracellular GSH depletion, and tightening constraints on GPX4-dependent detoxification (50–52). When elevated mtROS coincides with an expanded LIP, Fenton ignition accelerates self-propagating lipid peroxidation (6, 8). Under these conditions, mitochondria act as both sources of oxidative stress and targets of lipid and redox injury (11, 48).

Canonical mitophagy pathways, centered on the PTEN-induced kinase 1 (PINK1)/Parkin axis and related cargo receptors, remove damaged mitochondria to limit mtROS emission (11, 39, 53–55). In sepsis, this protective loop weakens as cellular energy declines and lysosomal degradation is compromised, allowing dysfunctional mitochondria to persist and accumulate (11, 48, 53). Mitophagy should be viewed as a key determinant of mtROS output, with direction and extent varying by stage (39, 54). Mechanistic interpretations in sepsis must be flux-aware: pair mtROS with mitochondrial turnover and lysosomal competence, avoiding reliance on static protein markers alone (11, 15).

### 3.4 p62–Keap1–Nrf2 signaling in redox proteostasis

Oxidative stress and ferroptosis are not purely chemical processes; they are tightly governed by signaling pathways (50). The p62–Keap1–Nrf2 axis, centered on Kelch-like ECH-associated protein 1 (Keap1) and nuclear factor erythroid 2-related factor 2 (Nrf2), serves as a major hub for integrating redox stress, autophagic proteostasis, and antioxidant responses (50–52).

Nrf2 coordinates the expression of antioxidant genes, supporting redox buffering capacity under oxidative stress (3). During sepsis, this critical signaling axis is dysregulated in a highly context-dependent manner. Insufficient activation of Nrf2-driven transcriptional networks renders cells more vulnerable to oxidative storm and accelerates depletion of endogenous antioxidant pools (53). This transcriptional failure accelerates GSH depletion, compromises lipid hydroperoxide detoxification, and increases susceptibility to ferroptosis (3, 55). Autophagy shapes the response: the autophagy receptor p62 sequesters Keap1, thereby promoting non-canonical Nrf2 activation (50–52). Through this mechanism, changes in overall degradative capacity and proteostasis directly influence the strength of antioxidant defenses, including ferroptosis resistance gates (50).

Beyond general antioxidant buffering, Nrf2 also regulates iron-handling genes for intracellular sequestration, export, and heme stress management (8, 22). This intersection is critical because labile iron drives the Fenton reaction in ferroptosis (8). The protective efficacy of these iron-handling programs is unpredictable in hyperoxic septic environments (3). This variability reflects differences in oxidant burden, heme stress, and lysosomal competence across experimental settings and disease stages; therefore, changes in transcripts alone rarely clarify whether the effect is protective or detrimental (8). To avoid overestimating signaling protection, interpretations require direct functional biochemical assays of LIPs and lipid peroxidation, rather than inference from Nrf2 target gene upregulation alone (55).

### 3.5 AMPK–mTOR signaling and autophagy

The AMP-activated protein kinase (AMPK) –mechanistic target of rapamycin (mTOR) axis functions as a central metabolic switch that integrates energy and nutrient signals to regulate autophagy (56, 57). During energy deprivation, AMPK activates autophagic clearance (57), whereas mTOR suppresses autophagy in nutrient-replete conditions, thereby maintaining metabolic homeostasis (56).

In sepsis, hypoxia, inflammatory stress, and metabolic collapse disrupt this regulatory balance (15, 40). As a result, autophagic flux becomes inefficient, lysosomal acidification is impaired, and degradative capacity declines (39, 40, 58). These changes indirectly promote ferroptosis. Defective autophagic clearance allows damaged mitochondria to accumulate (11, 58), sustain pools of peroxidation-prone lipid substrates (3), and disrupt intracellular iron metabolism (3). Together, these effects increase oxidative pressure on endogenous antioxidant defenses and promote lipid peroxidation (58).

Timing is also critical in this regard. Early in sepsis, oxidative bursts are essential for antimicrobial defense (59), while later stages are characterized by persistent redox injury and immunoparalysis (39, 59). Consequently, therapeutic strategies should not simply increase autophagy but should restore effective autophagic flux and lysosomal degradation capacity (40). Therapeutic strategies should also avoid inadvertently expanding the LIP or enriching membranes with peroxidation-prone lipids when oxidative stress has already been elevated (3).

### 3.6 Therapeutic targeting of autophagy–ferroptosis crosstalk

Taken together, autophagy–ferroptosis crosstalk in sepsis can be organized into three linked biochemical axes, shaped by two regulatory hubs (3, 6).

The iron axis modulates LIP and Fenton ignition, with ferritinophagy and lysosome-centered iron mobilization as primary routes (2, 6, 49). The lipid axis influences the availability of peroxidation substrates and shapes the conditions for chain propagation. It is driven largely by lipophagy and enzymatic remodeling (3, 22). Finally, the mitochondrial axis reflects how damaged mitochondria sustain mtROS output and amplify redox stress, while mitophagy acts as a key regulatory loop (11, 48, 49, 54). Operating alongside these axes are two hubs: the p62–Keap1–Nrf2 hub coordinates the transcriptional antioxidant response (50, 52), and the AMPK–mTOR hub governs energy-dependent autophagic flux and lysosomal competence (39, 57).

This mechanistic map provides a translational framework by identifying specific intervention nodes (3). Pharmacological interventions should be evaluated using axis-aligned biochemical markers, such as direct LIP quantification, lipid-ROS tracking, downstream malondialdehyde (MDA) and 4-hydroxynonenal (4-HNE) measurements, GPX4–GSH axis status, and mtROS/mitochondrial membrane potential (ΔΨm) assessments, rather than relying on single-protein narratives (2, 3, 55). Guided by this framework, antioxidant nanotherapeutics should be engineered to execute three primary strategies—neutralizing iron ignition, intercepting lipid propagation, and dampening mitochondrial amplification—while adhering to stage-specific deployment and compartment-restricted delivery.

## 4 Intelligent antioxidant nanotherapeutics

The current literature frequently classifies nanoplatforms based on their material composition (4, 10, 60). This section organizes antioxidant nanotherapeutics by their primary site at the autophagy–ferroptosis crossroads: iron ignition, lipid-peroxidation propagation, mitochondrial source control, and reinforcement of endogenous defense gates (3, 5, 9). The primary objective is clear: to connect material design with biological mechanisms (9, 10). We align delivery strategies with axis-specific biochemical markers to establish an assessment of therapeutic benefit and systemic risk (10).

**Figure 3:**
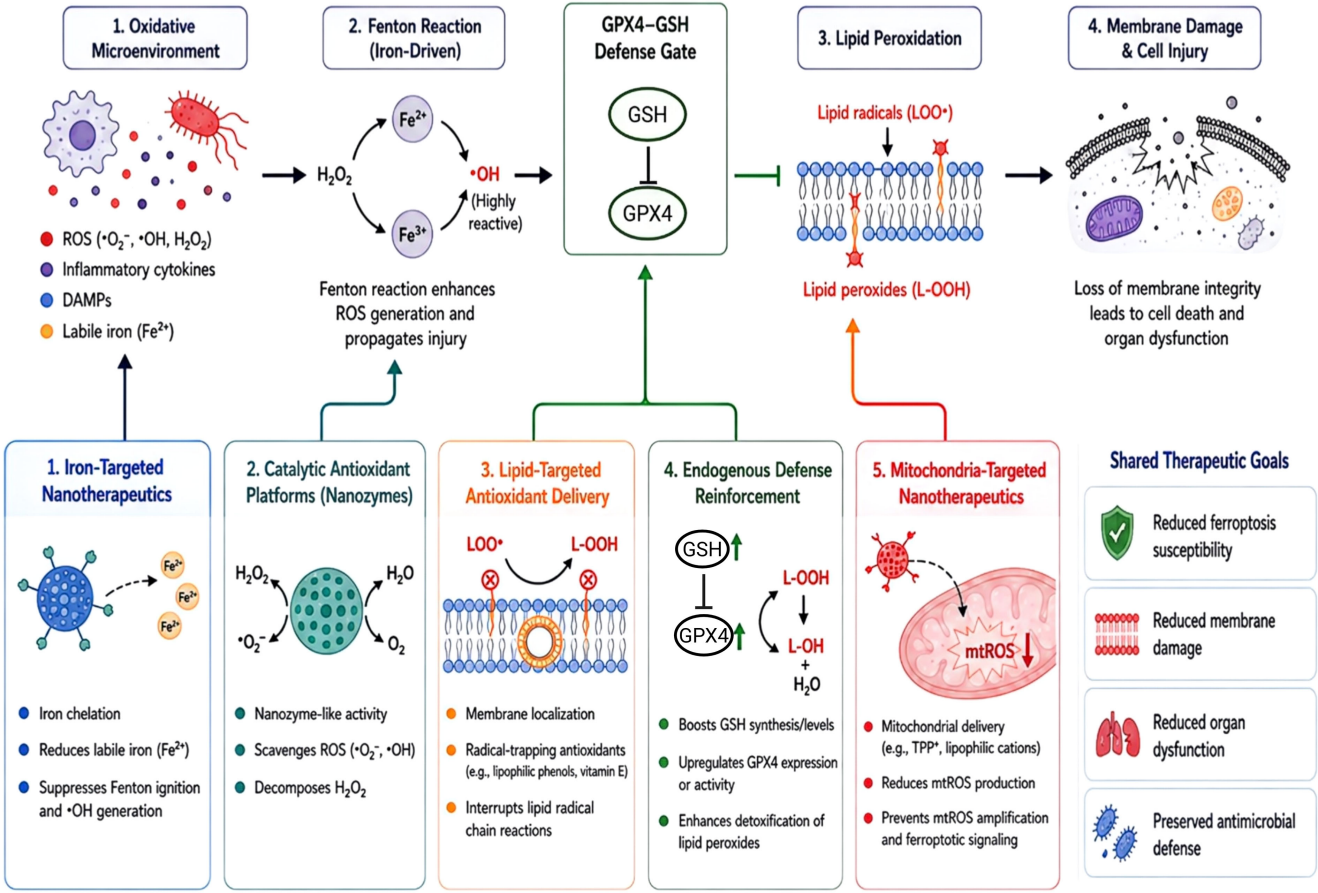
Mechanism-guided antioxidant nanotherapeutic strategies in sepsis. Mechanism-guided antioxidant nanotherapeutics can be organized according to the dominant biochemical nodes they target in sepsis-associated ferroptotic injury. In the septic oxidative microenvironment, iron-driven Fenton chemistry promotes reactive oxygen species (ROS) generation and initiates lipid peroxidation, while failure of the glutathione peroxidase 4–glutathione (GPX4–GSH) defense gate permits the accumulation of lipid peroxides and subsequent membrane damage. Iron-targeted nanotherapeutics reduce labile Fe²^+^ availability and suppress Fenton ignition. Catalytic antioxidant platforms (nanozymes) lower oxidant burden by scavenging ROS and decomposing H_2_O_2_. Lipid-targeted antioxidant delivery localizes radical-trapping agents to vulnerable membranes and interrupts lipid radical chain propagation. Endogenous defense reinforcement enhances GPX4–GSH related detoxification of lipid peroxides. Mitochondria-targeted nanotherapeutics reduce mitochondrial reactive oxygen species (mtROS) amplification and limit ferroptotic signaling. Together, these strategies aim to reduce ferroptosis susceptibility, preserve membrane integrity, and protect organ function while minimizing interference with antimicrobial defense. Created in BioRender. Huang, Y. (2026) https://BioRender.com/ic4xhr7.

Within the septic microenvironment, advanced nanoscale delivery exerts highly localized control over the intracellular redox environment (4, 10). This spatiotemporal precision is central to the sepsis “antioxidant paradox” (9, 60). It allows targeted nanomedicines to preserve localized oxidative bursts that are essential for early antimicrobial defense (2, 5, 10, 58). Nanotherapeutics should match specific disease stages and tissue compartments to fulfill two linked goals: organ protection and the preservation of host immune competence (10, 60). The major mechanism-guided antioxidant nanotherapeutic strategies discussed in this section are summarized in **Figure 3**.

### 4.1 Design principles and therapeutic evaluation

In sepsis, nanotherapeutics should be regarded as tools for localized redox control rather than simply “stronger antioxidants” (5, 9, 10, 60). Fenton ignition depends on local labile iron and H_2_O_2_ (3, 6), while mtROS arise from damaged mitochondria (11, 49). When oxidant pressure overwhelms endogenous defense gates—most prominently the GPX4–GSH axis (2, 3, 52, 55)—nanoplatforms can be designed to concentrate activity at these vulnerable sites (4, 9).

Each nanotherapeutic system should be categorized according to its primary node: iron ignition, lipid-peroxidation propagation, mtROS amplification, or gate reinforcement (3, 10, 60). Common approaches include local iron chelation or buffering, termination of lipid-radical chain reactions, catalytic removal of H_2_O_2_, and support for GSH-CoQ-BH_4_-centered defenses (5, 9, 10, 52). Many nanoplatforms influence more than one step, and cross-node effects are plausible (60, 61). However, multi-target claims should be made only when supported by aligned biochemical measures rather than inferred from survival curves or cytokine shifts alone (10).

Early infection-driven sepsis relies on oxidative bursts, including NOX-dependent responses, to support pathogen clearance (4, 10). Later phases often feature persistent oxidative injury, metabolic failure, and immunoparalysis (10, 60). Nanomedicine may help manage this boundary by narrowing exposure to vulnerable parenchymal tissues or selected subcellular sites, thereby reducing unintended suppression of immune oxidative programs (4, 9, 60).

### 4.2 Iron-targeted nanotherapeutics

Metal–phenolic networks and polyphenol-based platforms leverage coordination chemistry and radical buffering (62, 63). Their primary site of intervention for these platforms is the iron ignition axis (5). In sepsis, elevated H_2_O_2_ becomes particularly damaging when it coincides with an expanded LIP; by sequestering iron, these platforms reduce the conversion of H_2_O_2_ into •OH, lower ignition pressure, and limit lipid peroxidation initiation (3, 62, 63). By encapsulating these polyphenols into engineered nanocarriers, researchers can enhance their systemic stability, increase tissue accumulation, and precisely control their degradation behavior (64, 65).

Another point of intervention is the lysosome. Ferritinophagy and iron mobilization are lysosome-dependent processes, and lysosomal dysfunction reduces controlled iron release (3, 6). Recent preclinical studies have provided supporting evidence for this iron-centric strategy (61). Naturally derived MPNs, such as tannic acid (TA)-based platforms, and melanin-inspired polydopamine (PDA) nanoparticles have shown protective effects in various models of sepsis-induced organ injury, including acute kidney injury and systemic inflammation (66–68). These nanoplatforms frequently traffic toward the lysosomal compartment, representing an additional relevant site of intervention (69). By accumulating at this subcellular site, PDA- and TA-based platforms can locally sequester labile Fe²^+^, potentially raising the threshold for irreversible membrane damage (70, 71).

### 4.3 Catalytic antioxidant platforms

Nanozymes are often presented as enzyme-mimetic catalysts that perform SOD-, catalase (CAT)-, or peroxidase (POD)-like activities to buffer ROS in sepsis (9, 10, 72). Their primary therapeutic advantage in sepsis lies in buffering rather than complete elimination of ROS (10, 73). Oxidative stress biology is sustained by the generation of oxidants from mitochondria and NOX, which makes antioxidants susceptible to rapid depletion (3). Catalytic turnover can stabilize local redox conditions when buffering remains the dominant reaction mode (9, 72). This balance is microenvironment-dependent and changes with local pH, chloride and thiol availability, protein corona formation, and peroxide burden (10).

Mechanistically, upstream H_2_O_2_ buffering by nanozymes can suppress Fenton ignition, thereby relieving the oxidative burden on the endogenous GPX4–GSH defense system (3, 9, 10). This approach has a clear therapeutic boundary: once lipid peroxidation propagation is fully established, upstream catalytic buffering becomes insufficient, direct chain-breaking interventions may be required (3, 13, 20).

Transition metal oxide nanozymes—such as ceria- or manganese-based systems with combined SOD/CAT-like behavior—have been tested in sepsis models as local peroxide buffers (4, 10, 74, 75). Support for the proposed mechanism is stronger when reductions in ROS or H_2_O_2_ are matched by corresponding changes in ferroptosis-related markers. These should include lipid-ROS dynamics, terminal lipid-peroxidation products, and GPX4–GSH-related readouts in the target tissue (3, 53, 76). Because metallic nanozymes can absorb light or interfere with fluorescent probes, researchers should report optical assay-interference controls to avoid confounding measurements of ROS and lipid hydroperoxides (77).

### 4.4 Lipid-targeted antioxidant delivery

RTAs directly target ferroptosis by terminating lipid-peroxidation propagation in membranes (2, 3). They intercept lipid peroxyl radicals, prevent chain transfer, and limit the accumulation of lipid hydroperoxides (2).

In sepsis, this strategy is reasonable because oxidant sources are persistent and heterogeneous, and complete suppression of ROS generation may be neither feasible nor appropriate (2, 3). Once lipid-peroxidation propagation is established, reactive lipid intermediates can continue to drive membrane injury even when upstream oxidant pressure eases (2). The clinical application of RTAs, such as ferrostatin-1 and liproxstatin-1, is frequently limited by delivery challenges (3, 78). Their poor aqueous solubility and rapid systemic clearance often limit their accumulation within the hydrophobic membrane core, which is the primary site of lipid peroxidation (4, 9, 10, 78).

To overcome these delivery barriers, recent preclinical studies have incorporated hydrophobic RTAs into lipid-based and biomimetic nanocarriers (79–81). For example, liposomal formulations and macrophage-membrane-camouflaged nanoparticles have been used to deliver Fer-1 or Lip-1 in models of sepsis-induced acute lung and kidney injury (10, 75, 82, 83). These engineered vesicles can navigate the hyperinflammatory circulation and interact directly with the plasma membranes of injured cells (84, 85). In doing so, they embed RTA molecules within vulnerable lipid bilayers, where peroxyl radicals propagate (86, 87). Evaluation of these membrane-targeted platforms should be grounded in reductions of terminal lipid peroxidation markers, such as MDA and 4-HNE, together with preservation of membrane integrity, rather than relying solely on cytosolic ROS measurements (27, 88, 89).

### 4.5 Reinforcement of endogenous antioxidant systems

Gate-focused designs aim to prevent ferroptosis by supporting endogenous defense systems (2, 3). The GPX4–GSH axis is the primary gate (3, 27, 52), while auxiliary layers may contribute in specific compartments. In sepsis, oxidative stress and metabolic instability deplete antioxidant reserves (27, 52, 61). Gate reinforcement can be considered a threshold-shifting intervention that raises the oxidant pressure needed to trigger runaway lipid peroxidation (3, 88).

Nanoplatforms may reinforce this gate through several complementary strategies, including local oxidant buffering, cofactor delivery, and support of reductive capacity (3, 9, 10, 90). Another option is co-delivery: lowering local oxidant burden while replenishing reductive capacity in the same compartment (8, 9). These approaches change buffering capacity, so they should be evaluated with gate-facing readouts rather than general oxidative-stress markers (3, 27).

Recent preclinical studies have provided proof-of-concept support for this approach (10, 90). Selenium-based nanoplatforms and redox-active delivery systems have been explored in models of sepsis-associated organ dysfunction, particularly in settings involving oxidative stress, ferroptosis-related injury, and impaired GPX4–GSH defense capacity (3, 10, 90). These platforms reinforce the GPX4–GSH axis either by supporting GPX4-related activity or by replenishing intracellular GSH (3, 90, 91). By reinforcing this lipid-peroxide detoxification gate (52), they can increase cellular capacity to clear lipid hydroperoxides and temper downstream membrane injury (3, 91). However, mechanistic evidence should rely on gate-aligned readouts in the target tissue, including restoration of GSH redox status, GPX4-related measures, and reduced terminal lipid-peroxidation markers (26, 50, 91).

### 4.6 Mitochondria-targeted nanotherapeutics

Mitochondria-targeted strategies address a key source of oxidative stress in sepsis by disrupting the self-reinforcing loop of mtROS overproduction and lipid peroxidation (14, 48, 49). Engineering approaches using lipophilic cations, such as triphenylphosphonium (TPP^+^), or mitochondria-targeted peptides, such as Szeto–Schiller 31 peptide (SS-31), can direct redox-active nanocarriers toward the mitochondrial compartment (92, 93). Rather than acting as nonspecific antioxidants, these platforms are designed to attenuate mtROS amplification and relieve strain on the endogenous GPX4–GSH axis defense system (14). Mitochondria-targeted buffering may help reduce mitochondrial damage in sepsis by limiting local mtROS burden and stabilizing mitochondrial function (48, 94). However, reduced mtROS alone does not demonstrate restored mitophagy and should be interpreted together with flux-based evidence of mitochondrial clearance (11, 39, 54). Lower mtROS can stabilize mitochondria and create conditions more compatible with effective mitochondrial quality control (48).

TPP^+^-modified nanotherapeutics loaded with specific antioxidants have been investigated in models of sepsis-induced cardiomyopathy and acute kidney injury (93, 95, 96). Driven by ΔΨm, these platforms are designed to cross the double membrane and neutralize superoxide directly within the matrix (92, 95). By helping to preserve mitochondrial structural integrity and limit the release of pro-apoptotic factors, these systems target a key metabolic engine of oxidative injury (93, 96). Accordingly, the evaluation of such targeted nanotherapeutics should incorporate organelle-specific biochemical markers, such as the restoration of ΔΨm and the reduction of matrix superoxide, rather than relying solely on generalized oxidative stress indicators (92, 95, 96).

### 4.7 Therapeutic matching by disease stage and injury chemistry

In practice, therapeutic platform selection should follow the dominant injury chemistry and the compartment that is failing (10, 60). When biochemical evidence indicates an ignition-dominant setting, expanded LIP often coexists with elevated H_2_O_2_ (3, 52). In this context, iron sequestration or chelation, together with catalytic peroxide buffering, provides the most direct way to reduce Fenton reactivity (4, 8). When mitochondrial dysfunction acts as an amplifier—bioenergetic failure alongside mitochondrial injury measures—mitochondria-targeted mtROS control is the most direct way to dampen this feed-forward loop (4, 11, 48). By contrast, ferroptosis vulnerability may be driven mainly by failure of endogenous antioxidant defenses. When GPX4 function is impaired or GSH is depleted, strategies that restore lipid-peroxide detoxification may be the most appropriate choice (3, 26, 91).

Timing adds a second level of decision-making. Early in sepsis, oxidative bursts remain part of the antimicrobial defense; therefore, redox intervention benefits from tighter targeting and stage-aware timing to avoid weakening host clearance (10, 60). Targeted platforms can preferentially accumulate in vulnerable parenchymal tissues while limiting off-target systemic exposure (75, 94).

## 5 Organ and immune outcomes

Oxidative storm chemistry is systemic, ferroptotic susceptibility is compartment-dependent (3, 11, 50). Oxidant pressure does not translate into uniform injury. Instead, whether a tissue tips into ferroptosis-relevant damage depends on its intrinsic biochemical constraints, including local metabolism, iron-handling capacity, membrane lipid composition, and mitochondrial density (3, 11, 97). Some compartments are relatively iron-primed, with an increased tendency to expand the LIP (27). Others are lipid-primed or mitochondria-primed (5, 11).

Perfusion defects, hypoxia, and inflammatory mediators remodel the local microenvironment and shift the dominant chemistry (11). As a result, the same redox-directed nanotherapeutic can help one compartment while harming another (10, 48).

The clinical value of the crossroads framework depends on how precisely it connects mechanisms to compartments and stages (97, 98). Broad labels, such as “oxidative stress, “ are insufficient; what matters is whether the framework can explain organ-specific failure patterns and immune compromise (27, 98). Therefore, we have focused on compartment-resolved evidence rather than circulating markers alone (50). Across organs, we have connected vulnerabilities with the iron–lipid–mitochondria axes and laid out the biochemical panels that best support ferroptosis-relevant injury (3). We have then made timing explicit by defining stage-aware boundary conditions that balance tissue protection with the preservation of immune competence (27).

### 5.1 Kidney injury and ferroptosis susceptibility

The kidney is particularly vulnerable in sepsis (3, 50, 99). In the iron–lipid–mitochondria crossroads framework, kidney vulnerability stems from three main drivers: strong dependence on mitochondrial ATP production and sensitivity to mitochondrial dysfunction and mtROS amplification (14, 48, 100); rapid loss of membrane integrity when lipid-peroxidation propagation accelerates under strained antioxidant defense (50, 101); and heightened susceptibility to iron-driven ignition when the LIP expands during inflammatory or heme stress. These conditions, identifying ferroptotic injury, requires an integrated, axis-spanning biochemical panel rather than a single marker (3, 50).

Rather than relying on generic indicators of oxidative capacity, evaluating the iron axis requires measuring the chelatable iron pool and tracking lysosomal iron mobilization (99, 102). For the lipid axis, dynamic lipid-ROS tracking should be assessed through terminal lipid peroxidation products, such as MDA or 4-HNE (3, 99). Furthermore, the functional integrity of the primary antioxidant gate should be reflected by the GSH/oxidized glutathione (GSSG) redox state and GPX4 activity, rather than static protein expression (3, 26, 50). To capture the mitochondrial contribution, mtROS quantification can be combined with ΔΨm to confirm redox amplification (54, 95). Crucially, if autophagy is implicated, researchers must measure dynamic autophagic flux; simply observing an accumulation of autophagosomes often indicates blocked lysosomal clearance rather than a successful protective response (54, 103). To confirm true organ failure, these molecular markers should be tightly correlated with clinical renal function and tubular damage (3, 99).

Translating these pathophysiological mechanisms into nanomedicine design involves matching the therapeutic platform to the specific biochemical vulnerability within the renal microenvironment. Mitochondria-targeted nanocarriers may reduce tubular injury by buffering oxidants at their mitochondrial source and stabilizing local bioenergetic function (48, 95). At the plasma membrane, RTAs halt ferroptotic execution by intercepting lipid-radical propagation (104). Upstream peroxide pressure can be buffered by catalytic nanozymes to relieve the oxidative burden on the GPX4–GSH axis (105), as localized iron chelation becomes appropriate when labile iron expansion is biochemically triggered (3, 102). Beyond localized rescue, therapeutic deployment is governed by strict immune boundary conditions: targeted distribution secures renal protection without broadly suppressing antimicrobial oxidative bursts during early sepsis (10, 60).

### 5.2 Pulmonary barrier dysfunction and lipid peroxidation

The pulmonary compartment is inherently lipid-primed because gas exchange depends on extremely thin endothelial and epithelial bilayers (8). In sepsis, the rapid accumulation of lipid peroxides within fragile membranes directly disrupts the alveolar-capillary barrier, leading to increased microvascular permeability, exudation, and edema (8, 88). This damage is further intensified by inflammatory cell infiltration. Neutrophils and macrophages release concentrated ROS and RNS, which interact with local iron pools to drive lipid peroxidation and barrier failure (8, 53).

Ferroptosis in the pulmonary barrier requires an integrated approach rather than relying on single markers (8). Assessments should combine membrane-centric biochemical markers, such as dynamic lipid-ROS and terminal lipid peroxidation products, with functional barrier permeability assays (8, 88). The functional status of the GPX4–GSH axis defense gate should also be evaluated to estimate endogenous buffering capacity of the lung (8, 88). The interpretation of autophagy in the pulmonary barrier requires flux-based assessment, because accumulation of autophagosomes in alveolar cells may reflect impaired lysosomal clearance rather than effective protection (22).

This non-invasive route directly addresses the systemic antioxidant paradox (1, 10). It allows RTAs or catalytic nanozymes to achieve higher local concentrations at inflamed alveolar surfaces while limiting unnecessary systemic exposure (10, 48, 97). By limiting lipid-peroxidation damage within the pulmonary barrier, such strategies may help preserve lung integrity while minimizing unnecessary systemic exposure (48, 97).

### 5.3 Cardiac dysfunction and mitochondrial redox injury

Sepsis-induced cardiomyopathy is particularly susceptible to mtROS amplification (15, 106, 107). Cardiomyocytes maintain a high mitochondrial density to meet massive ATP demands (15). In sepsis, microvascular hypoperfusion and circulating inflammatory mediators rapidly disrupt this delicate architecture and cause severe energy deficits (15, 106). Damaged mitochondria then become primary generators of mtROS (107, 108). This localized oxidative stress can deplete regional GPX4–GSH axis antioxidant reserves, thereby promoting lipid peroxidation and myocardial dysfunction (3, 108, 109). Although iron dysregulation may contribute to early cellular stress (107, 110), mitochondria-directed suppression of mtROS often represents the most direct strategy for limiting myocardial redox amplification and preserving cardiac function (48, 93).

Evaluation of cardiac ferroptosis should therefore integrate mitochondrial readouts, terminal lipid-peroxidation markers, and functional measures of cardiac performance (3). In practice, mtROS and mitochondrial ΔΨm should be assessed together with MDA or 4-HNE and measures of cardiac output (3, 107). Taken together, these markers help distinguish ferroptotic progression from transient metabolic strain (108).

For cardiac nanomedicine, the main engineering challenge is achieving effective subcellular delivery to mitochondria (48, 92, 93). Conventional small-molecule antioxidants typically fail to reach the mitochondrial matrix and may suppress early-phase antimicrobial defenses (4, 10, 48). To address this limitation, nanocarriers can be modified with lipophilic cations, such as TPP^+^, or with mitochondria-targeting peptides, thereby promoting mitochondrial delivery of catalytic nanozymes or RTAs (92, 93, 96, 111). By reducing superoxide at its source, these systems may help break the self-reinforcing cycle of mitochondrial redox injury while preserving broader immune function (93, 96).

### 5.4 Immune reprogramming, ferroptosis, and immunoparalysis

Integrating the ferroptosis framework into the immune compartment introduces an important biological constraint in sepsis (2, 27). Unlike parenchymal tissues, immune cells intrinsically rely on ROS and RNS to execute antimicrobial defense (27). During the early stages of sepsis, macrophages and neutrophils generate robust oxidative bursts that are essential for pathogen clearance (53). However, when oxidative stress becomes persistent and poorly controlled, these redox mechanisms can push immune cells toward regulated cell death pathways (2).

In macrophages, susceptibility to ferroptosis is shaped by the balance between intracellular iron handling, mitochondrial quality control, and the integrity of the GPX4–GSH axis antioxidant system (3, 27). When lipid peroxidation exceeds the buffering capacity of this defense gate, macrophages progressively lose their effector functions and may release damage-associated molecular patterns, amplifying inflammatory signaling and tissue injury (2, 61).

The adaptive immune compartment faces a related but distinct vulnerability. T cells depend heavily on metabolic fitness and mitochondrial integrity to sustain activation and proliferation (3). Persistent ROS accumulation and depletion of intracellular glutathione disrupt this balance, promoting T-cell dysfunction, exhaustion, and ferroptotic susceptibility (2, 3). These dynamics have important implications for experimental evaluation and therapeutic design. Mechanistic studies should prioritize functional immune competence rather than relying solely on cell survival. Measurements of ROS burst capacity and T-cell functional responses should be interpreted in conjunction with ferroptosis-related markers, including lipid-ROS dynamics and terminal lipid peroxidation products, such as MDA or 4-HNE (3, 112).

For nanomedicine-based redox interventions, the immune compartment imposes a strict boundary condition (9, 10). Protective approaches must reduce pathological lipid peroxidation within vulnerable immune subsets while preserving the reactive-species signaling required for early antimicrobial defense (9).

### 5.5 Systemic integration and the antioxidant paradox

Redox injury in sepsis is not confined to individual cells or isolated organs; rather, it spreads across interconnected organs and immune systems, making both diagnosis and targeted therapy more difficult (1). For example, the liver acts as a central hub for systemic iron metabolism and macrophage-rich immune surveillance (113). During the acute-phase response, changes in circulating iron indices may not reflect true intracellular labile iron dynamics, making direct tissue-based biochemical assessment important for confirming hepatic ferroptosis (3, 42, 55). At the same time, the gut barrier serves as an important amplifier of systemic vulnerability. Excessive oxidative pressure disrupts epithelial barrier integrity and promotes lipid peroxidation, thereby sustaining systemic inflammation through the gut-liver axis (114, 115). The neurovascular unit adds another layer of complexity: in sepsis-associated encephalopathy, effective neuroprotection requires crossing the blood–brain barrier without compromising neuroimmune safety (116, 117). Together, these overlapping systemic vulnerabilities and the need to balance organ protection with immune preservation define the antioxidant paradox in sepsis: reactive species are not universally detrimental (1, 5). Traditional non-targeted antioxidants have shown limited benefit because they may indiscriminately suppress antimicrobial oxidative programs required for early host defense, yet still fail to achieve sufficient concentrations at sites of ferroptotic tissue injury (4, 118). Resolving this paradox requires viewing redox modulation as a spatiotemporal and compartment-specific problem (10). The translational goal of advanced nanotherapeutics is not maximal ROS scavenging, but selective and precise redox control (66, 119). By engineering platforms for compartment-restricted delivery—whether organ-targeted, membrane-camouflaged, or mitochondria-directed—nanomedicines may limit lipid-radical propagation and buffer oxidant stress at the sites where injury accumulates (10, 48, 60). If guided by mechanism-based biomarkers and clear biosafety considerations, such strategies may offer a realistic path toward organ protection without compromising host immune competence.

### 5.6 Translational challenges and strategic priorities

Despite growing mechanistic clarity, translating autophagy–ferroptosis-directed redox nanomedicine into clinical practice remains challenging (3, 5, 98). Patient heterogeneity, dynamic disease staging, and compartment-specific injury chemistry mean that the same intervention may be protective in one context and detrimental in another (2, 120). Effective translation requires precise subcellular delivery, pharmacokinetic competence, and preservation of the oxidative capacity necessary for host antimicrobial defense (4, 10, 94, 119). Mechanistic validation should also rely on aligned biochemical evidence, such as LIP dynamics, lipid peroxidation burden, GPX4–GSH status, and mtROS-related stress (3, 5, 26, 48). Although clinically meaningful, survival benefit alone should not be taken as sufficient evidence that the intended redox target has been modulated (120). Finally, long-term biosafety, scalable manufacturing, and regulatory feasibility remain unresolved for many nanoplatforms (119, 121, 122). Taken together, further progress will require not only deeper mechanistic insight but also careful target validation, precise delivery, and realistic translational planning (120–122).

Several practical issues require more explicit consideration before antioxidant nanomedicine can move toward clinical application in sepsis (123). These translational barriers are closely interconnected and limit the transition from preclinical proof-of-concept to clinically applicable therapy (123). Targeting efficiency remains difficult to sustain in the heterogeneous septic microenvironment, where altered perfusion, endothelial leakage, and rapidly evolving organ injury can limit predictable tissue and subcellular delivery (16, 17, 123). To address this challenge, biomimetic strategies—such as coating nanocarriers with macrophage or neutrophil membranes—may improve circulation compatibility and enhance accumulation in inflamed or injured tissues (82). Large-scale and reproducible manufacturing is another major obstacle for multifunctional nanoplatforms that require precise control of physicochemical properties and batch consistency (123–125). Possible solutions include simplifying nanoplatform design and adopting more scalable preparation methods, such as automated or microfluidic-assisted manufacturing, which may improve batch-to-batch consistency and translational feasibility (123–125). Furthermore, long-term biodistribution, retention, and biosafety remain insufficiently evaluated in critically ill hosts, and cost-effectiveness may become a decisive barrier in emergency and intensive care contexts (123, 126). To mitigate these limitations, future development should prioritize biodegradable and clinically translatable material systems, including endogenous, naturally derived, or already approved building blocks (123, 126). The major challenges and corresponding strategic priorities are summarized in **Table 1**.

**Table 1:** Major translational challenges and strategic priorities for precision redox nanomedicine in sepsis.

Dimension	Focus	Limitations	Barriers	Priorities
Patient selection	Biomarker- and stage-guided therapy	High heterogeneity and limited specificity of current markers.	Poor stratification dilutes treatment effects.	Use biomarker-guided, stage-aware selection (2, 120).
Targeting and pharmacokinetics	Organ and compartment delivery	Poor solubility, rapid clearance, and weak target accumulation.	Off-target exposure lowers efficacy.	Improve targeting and pharmacokinetic stability (4, 10, 94, 119).
Immune boundary	Preserve antimicrobial defense	Broad antioxidant effects may suppress host defense.	Timing and scope of therapy are restricted.	Use stage- and compartment-restricted strategies (5, 10, 120).
Mechanistic validation	Confirm target engagement	Static markers alone are insufficient.	Weak mechanistic evidence limits translation.	Use aligned biochemical markers and flux-aware assays (3, 5, 26, 48).
Safety and regulation	Biosafety and GMP feasibility	Toxicity, retention, and scale-up issues remain unresolved.	Regulatory uncertainty slows translation.	Prioritize scalable materials and extended biosafety testing (119, 121, 122).

## 6 Conclusions and future perspectives

Sepsis pathogenesis extends beyond pathogen burden and reflects a profound breakdown of host redox homeostasis (1, 32). During sepsis, an oxidative storm promotes ferroptotic injury in vulnerable organs (3, 32). At the molecular level, this process is strongly influenced by autophagy-dependent pathways. Ferritinophagy, lipid remodeling, and mitophagy influence cellular susceptibility by regulating labile iron availability, the abundance of peroxidation-prone membrane substrates, and mtROS amplification (3, 39, 54). Autophagy–ferroptosis crosstalk is therefore not simply a parallel stress response; it helps determine whether the host response remains adaptive or shifts toward self-sustaining tissue injury in sepsis (3, 39).

Future progress will depend less on indiscriminate ROS suppression and more on selective control of redox injury (1, 32). A central challenge is to resolve the antioxidant paradox through stage-aware and compartment-specific strategies that protect vulnerable membranes and organelles without compromising antimicrobial oxidative programs required for host defense (1, 27, 120). Translating these strategies into clinical practice requires biomarkers that reflect the underlying biochemistry of injury. Such biomarkers should track iron status, including LIP-related signals, lipid-ROS dynamics, and downstream lipid peroxidation products, as well as mitochondrial stress markers such as mtROS and ΔΨm (3, 48, 120). Together, they may support patient stratification and help determine when and where intervention is most appropriate (120). Overall, the autophagy–ferroptosis crossroads provides a mechanism-based framework for evaluating redox-directed therapies and guiding the development of precision nanomedicine for organ protection in sepsis (27, 98, 119, 120).
